# Current status of infection with infectious hypodermal and hematopoietic necrosis virus (IHHNV) in the Peruvian and Ecuadorian shrimp industry

**DOI:** 10.1371/journal.pone.0272456

**Published:** 2022-08-10

**Authors:** Luis Fernando Aranguren Caro, Muriel Maria Gomez-Sanchez, Yahira Piedrahita, Hung Nam Mai, Roberto Cruz-Flores, Rod Russel R. Alenton, Arun K. Dhar

**Affiliations:** 1 Aquaculture Pathology Laboratory, School of Animal and Comparative Biomedical Sciences, The University of Arizona, Tucson, Arizona, United States of America; 2 Subdireccion de Sanidad, Dirección de Sanidad e inocuidad, National Fisheries Health Agency in Peru (SANIPES), San Isidro, Lima, Perú; 3 Camara Nacional de Acuacultura, CNA, Avenida Francisco de Orellana y Miguel H Alcivar, Guayaquil, Ecuador; 4 Centro de Investigación Científica y Educación Superior de Ensenada (CICESE), Ensenada, Baja California, México; Sathyabama Institute of Science and Technology, INDIA

## Abstract

Infection with infectious hypodermal and hematopoietic necrosis virus (IHHNV) is a crustacean disease that caused large-scale mortality in *Penaeus stylirostris*, deformity and growth retardation in *Penaeus vannamei* and *Penaeus monodon*. We surveyed the presence of IHHNV in three major shrimp-producing regions in Ecuador, namely Guayas, El Oro, and Esmeralda. The data show that IHHNV is endemic (3.3–100% prevalence) to shrimp farms in these regions. The whole genome sequences of representative circulating IHHNV genotypes in Ecuador and Peru showed that these genotypes formed a separate cluster within the Type II genotypes and were divergent from other geographical isolates of IHHNV originating in Asia, Africa, Australia, and Brazil. In experimental bioassays using specific pathogen-free (SPF) *P*. *vannamei*, *P*. *monodon*, and *P*. *stylirostris* and representative IHHNV isolates from Ecuador and Peru, the virus did not cause any mortality or induce clinical signs in any of the three penaeid species. Although IHHNV-specific Cowdry type A inclusion bodies were histologically detected in experimentally challenged *P*. *vannamei* and *P*. *monodon* and confirmed by *in situ* hybridization, no such inclusions were observed in *P*. *stylirostris*. Moreover, *P*. *vannamei* had the highest viral load, followed by *P*. *monodon* and *P*. *stylirostris*. Based on IHHNV surveillance data, we conclude that the currently farmed *P*. *vannamei* lines in Ecuador are tolerant to circulating IHHNV genotypes. The genome sequence and experimental bioassay data showed that, although the currently circulating genotypes are infectious, they do not induce clinical lesions in the three commercially important penaeid species. These findings suggest a potentially evolving virus-host relationship where circulating genotypes of IHHNV co-exist in equilibrium with *P*. *vannamei* raised in Peru and Ecuador.

## Introduction

Infectious hypodermal and hematopoietic necrosis virus (IHHNV) has been listed as a notifiable crustacean pathogen by the World Organization for Animal Health since 1995 [1] and is highly prevalent in America, Asia, and Australia. The virus previously caused large-scale mortalities in Pacific blue shrimp (*Penaeus stylirostris*) [2] and is known to cause growth retardation, described as runt-deformity syndrome (RDS), in Pacific white shrimp (*Penaeus vannamei*) [2, 3] and black tiger shrimp (*Penaeus monodon*) [4, 5].

IHHNV is a non-enveloped virus measuring 22–23 nm in size, and it contains a single-stranded DNA genome of ~ 4.1 kb in length [6]. IHHNV is classified as “*Decapod penstylhamaparvovirus 1*” in the family *Parvoviridae* and sub-family *Hamaparvovirinae* [7]. Parvoviruses have been shown to have a high mutation rate, similar to RNA viruses [8]. The nucleotide substitution rate of IHHNV was estimated at 1.39 × 10^−4^ substitutions/site/year [9]. Therefore, the occurrence of novel genotypes in different shrimp farming regions is unsurprising; currently, the presence of five genotypes (three infectious types: I, II, and III, and two non-infectious types: A and B) of IHHNV has been documented [10].

IHHNV is an endemic pathogen in most Latin American countries including Ecuador, Mexico, Honduras, Colombia, Brazil, and Peru [11]. Infection with IHHNV has been reported in several life stages of *P*. *vannamei* including post-larvae, juveniles, and broodstock, without any clinical signs, including RDS [12–14]. At the farm level, the prevalence of the virus can range between 10% to 50%, and the animals may appear healthy. However, it remains unknown whether the presence of the virus makes shrimp more susceptible to other pathogens and whether chronically infected animals have an impact on growth and farm productivity. In 2019, Sellars et al. from Australia reported reduced growth performance of *P*. *monodon* infected with IHHNV, indicating that IHHNV remains an economically important viral pathogen in shrimp aquaculture [4]. The authors claimed that despite causing a benign infection, when high-load IHHNV-infected *P*. *monodon* post-larvae were used to stock ponds, the growth, general health, and survival of the shrimp, as well as the harvest yields of the ponds, were compromised [4].

*Penaeus monodon* and *P*. *vannamei* shrimp containing integrated IHNV-genome sequences, commonly known as endogenous viral elements (EVE), have been reported [15, 16]. EVE-containing *P*. *monodon* and *P*. *vannamei* shrimp do not produce infectious virions and hence cannot transmit the virus horizontally to non-infected hosts [16–18]. Furthermore, the presence of EVE can be considered beneficial because it can give rise to small interfering RNA (siRNA) involved in viral inhibition via the RNA interference (RNAi) pathway [19].

Updates on the current impact of IHHNV on the Ecuadorian and Peruvian shrimp industry is insufficient to determine the pathogen impact at the farm level. According to the OIE, however, in years 2012–2019, there have been total of 25 and 21 cases reported of IHHNV in grow out ponds in Ecuador and Peru, respectively [1]. The objective of this study was to determine the influence of the circulating infectious strains(s) of IHHNV present in Peru and Ecuador on shrimp production in these two countries using three different approaches. These include 1) determining the prevalence of IHHNV and assessing the effect of IHHNV on shrimp growth in commercial grow-out ponds; 2) determining the full-length genome sequence of IHHNV genotypes in Ecuador and Peru and examining their relationship to other IHHNV isolates reported elsewhere in the world; and 3) assessing the infectivity of Peru and Ecuador isolates of IHHNV on three different penaeid species, *P*. *vannamei*, *P*. *monodon*, and *P*. *stylirostris*.

## Materials and methods

### Samples and sampling location

**Fig 1:**
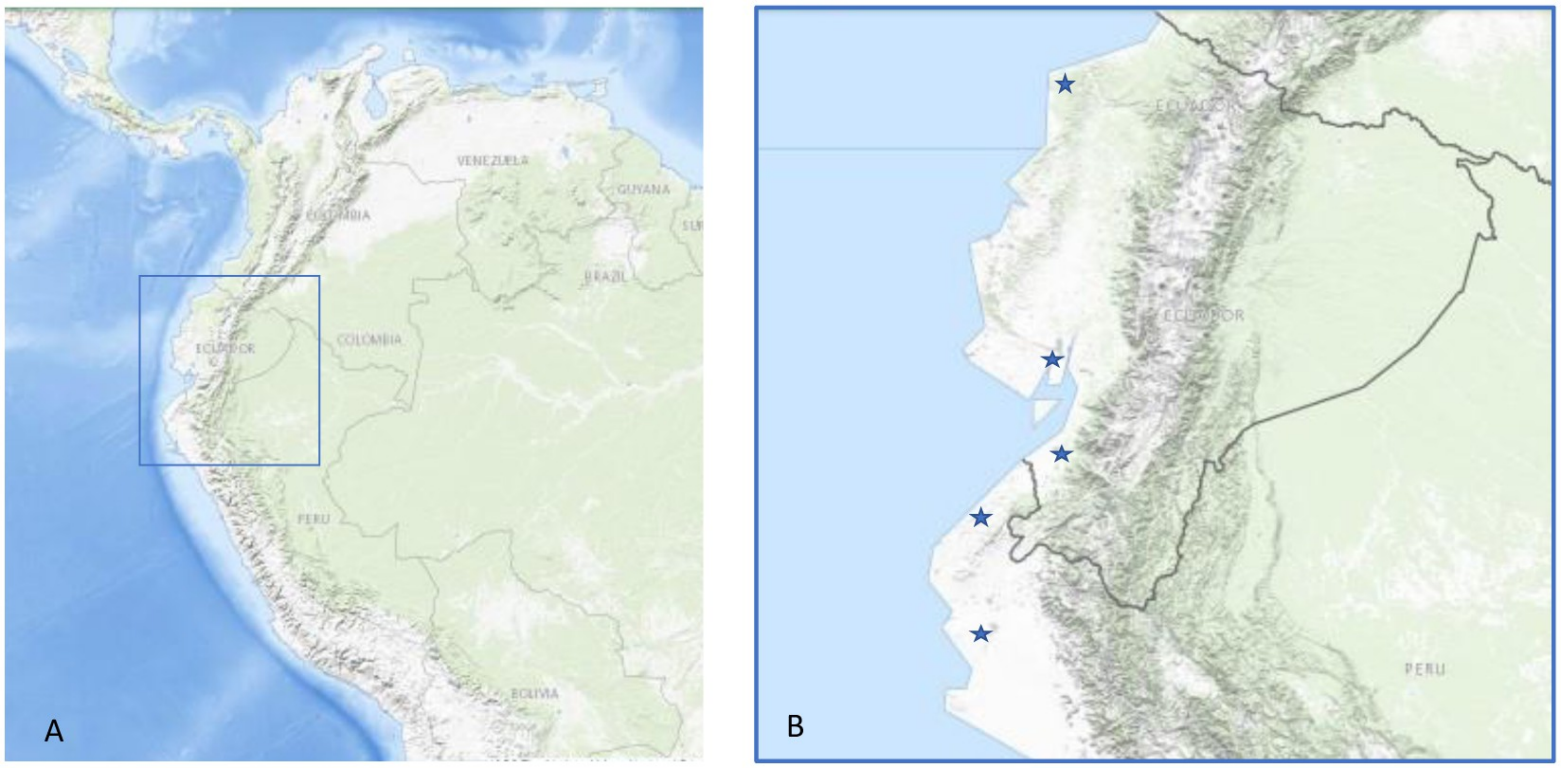
Location of the region in South America where IHHNV samples were obtained (A). Higher magnification of the boxed area in A showing sampling areas indicated by a star (★) symbol (B). The corresponding map is acquired from USGS National Map Viewer (http://viewer.nationalmap.gov/viewer/) for illustrative purposes only.

The IHHNV isolates were collected from Tumbes and Piura, north of Peru, and three regions in Ecuador, namely El Oro, Guayaquil, and Esmeraldas (Fig 1).

Sampling was conducted by the National Fisheries Health Agency in Peru (SANIPES) and the Camara Nacional de Acuacultura in Ecuador. Six IHHNV isolates from Peru were collected between 2019 and 2020 from shrimp farming regions in Tumbes (5): (3.48° S, 80.36 ° W; 3.46° S, 80.27 ° W; 3.45° S, 80.30 ° W; 3.47° S, 80.33 ° W; 3.52° S, 80.39 ° W)and Piura (1) (5.12° S, 80.58 ° W). In Ecuador, grow-out ponds displaying a high coefficient of variation (CV%>20) with a history of IHHNV occurrence were selected in 2020. Eight grow-out ponds distributed in Guayas (5), El Oro (1), and Esmeraldas (2) were analyzed. From each grow-out pond, 30 shrimp were randomly sampled, weighed, and had their pleopod and gill tissues preserved in 95% ethanol for real-time PCR analysis. Additional shrimp collected from each of these locations were individually frozen (−20°C) for further analysis. Finally, out of the same pond, 5 to 10 shrimp were fixed in Davidson’s fixative for histological analysis [20]. Samples from Peru and Ecuador were sent to the University of Arizona Aquaculture Pathology Laboratory (UA-APL) for further analysis.

### IHHNV detection and genome sequencing

#### DNA extraction

DNA extraction was performed individually on each sample using the Qiagen DNeasy Blood and Tissue Kit, following the manufacturer’s protocol. Following extraction, the DNA was stored at −20°C until further analysis.

#### Real-time PCR

The OIE-recommended method [21] was used for IHHNV detection and quantification. The nucleotide sequences of the primers used were IHHNV1608F: 5′-TAC TCC GGA CAC CCA ACC A-3′ and IHHNV1688R: 5′-GGC TCT GGC AGC AAA GGT AA-3′, and the TaqMan probe sequence was IHHNV-P1: 5′-[FAM] ACC AGA CAT AGA GCT ACA ATC CTC GCC TAT TTG-[TAMRA]-3′ [1]. The amplification reactions contained 0.5 μM of each primer, 0.1 μM TaqMan probe,1X TaqMan Fast virus 1-step Master Mix (Life Technologies, Carlsbad, CA. USA), 20 ng of DNA, and HPLC water in a reaction volume of 10 μl. The thermal cycling profile consisted of 20 s at 95°C, followed by 40 cycles of 1 s at 95°C and 20 s at 60°C. Amplification detection and data analysis for real-time PCR assays were carried out using a StepOnePlus real-time PCR system (Life Technologies, USA).

#### Whole genome sequencing

DNA extracted from samples (six from Peru, A, C, D, E, F, and G; and one from Ecuador, E1) that tested positive by real-time PCR were sent for next-generation sequencing (NGS). Library preparation was performed using TruSeq Stranded Total DNA Library Prep (Illumina, San Diego, CA. USA). Sequencing was performed using an Illumina HiSeq 2500 System (PE 2X150 bp) (Illumina ^®^, USA). The library for the DNA samples was generated at OmegaBioservices using the Library Kit KAPA Hyper prep for WGS (Roche, Germany).

The DNA reads generated from NGS were paired, and low-quality reads and adapters were trimmed prior to mapping to the IHHNV reference genome (GenBank accession number: AF218266) using Geneious Prime (Biomatters, New Zeland). Geneious mapper was used for mapping analysis, and the parameters were set to detect structural variants. All other parameters were set to their default values. The final IHHNV contig was edited manually. Annotation of the reference strain was used to annotate the IHHNV contigs.

### Phylogenetic analysis

The nucleotide sequences of 7 IHHNV isolates (6 from Peru and 1 from Ecuador) and 18 known IHHNV isolates obtained from the GenBank database were aligned using MAFFT (Auto: according to data size, 200 PAM, k = 2) (Geneious Prime, v2019). The best model for the phylogenetic tree was estimated using jmodel Test 2.1.10 [22]. A phylogenetic tree was constructed using MrBayes (http://www.phylogeny.fr/one_task.cgi?task_type=mrbayes) (KY85, 4by4, invariable+Gramma, number of generations = 10,000). The numbers indicate the posterior probability. In addition, the deduced amino acid sequences of the capsid protein from IHHNV isolates were aligned using MAFFT (Auto: according to data size, 200 PAM, k = 2), and the best model for the phylogenetic tree was estimated using ProtTest 3.4.2 [23]. A phylogenetic tree was constructed using MrBayes software (http://www.phylogeny.fr/one_task.cgi?task_type=mrbayes).

### Terminal repeats and palindromic sequence analysis

Two sequences (Peru D and C) generated contigs that exceeded the length of the largest known IHHNV sequence in the NCBI database. These two sequences are ~4000 nt long and present a unique opportunity to analyze the direct terminal repeats (DTR) that contain palindromic sequences forming hairpin-like structures. The DTR were manually curated using Geneious Prime, and ambiguities in the sequences were resolved by assigning the nucleotide that presented the largest proportion in a particular nucleotide position [24]. The terminal repeats were identified with the Geneious Prime repeat finder plug-in by adjusting the default parameters to identify repeats over 20 nt and allow four mismatches. Putative hairpin structures were modeled using the ProbKnot web server [25].

### IHHNV challenge test

Two independent IHHNV challenge tests were performed at the University of Arizona Aquaculture Pathology Laboratory (APL) in 2020 and 2021. Three different specific pathogen-free (SPF) juvenile penaeid shrimp species were used for these challenge tests (Table 1). *Penaeus vannamei* and *P*. *monodon* were obtained from vendors in the USA, and *P*. *stylirostris* was obtained from a vendor in Austria (White Panther and Shrimp Improvement Systems, originally from Mexico). The average weight of juveniles of each of these three species were 1.5±1.0 g. Twenty animals were stocked in a 90-L glass aquaria. In the tanks used to rear shrimp, temperature was maintained at 27.5±1.1°C, salinity at 28.3±1.1 ppt, pH 7.9±0.1, NH_3_ 0.05±0.1 ppm, and NO_2_ 0.05±0.1 ppm. The shrimp were fed once a day with a commercial pelleted feed (Rangen 35%; Buhl, Idaho). The details of the experimental design are presented in Table 1.

**Table 1:** Experimental designs of two independent IHHNV challenge tests using three different penaeid shrimp species.

Challenge test number	Origin of IHHNV isolate	Species	Treatment	Number of tanks	Number of shrimp / tank
1	Peru	*P*. *vannamei*	Control	1	20
		*P*. *vannamei*	IHHNV	3	20
2	Ecuador	*P*. *vannamei*	Control	1	20
		*P*. *vannamei*	IHHNV	3	20
		*P*. *monodon*	Control	1	20
		*P*. *monodon*	IHHNV	3	20
		*P*. *stylirostris*	Control	1	20
		*P*. *stylirostris*	IHHNV	3	20

Two different IHHNV inocula were prepared for the challenge test: one from Peru (Tumbes, Case 20-842F) and one from Ecuador (Guayas, 21–273). Frozen IHHNV-infected *P*. *vannamei* cephalothorax were homogenized in a buffer (0.02 M Tris-HCl, pH 7.4, 0.4 M NaCl buffer; 1 g 10 mL^−1^) using a tissue blender and clarified at 3500 × *g* for 20 min and at 5000 × *g* for 20 min at 4°C, respectively. The supernatant was filtered using a 0.2-μm filter, diluted (1:20) in 2% saline, aliquoted, and frozen at −80°C. The shrimp were injected with 100 μL of IHHNV. The viral load in the inoculum used to inject each animal was calculated as 9.0 × 10^6^ IHHNV genome copies. Mortality was recorded daily from the start of the experiment. At the end of the challenge test (i.e., 39 days post-inoculation, d.p.i) and at 30 d.p.i for Challenges 1 and 2, respectively, five survivors from each tank were individually sampled for histology and real-time PCR analyses. Gill samples were individually collected in 95% ethanol for real-time PCR analysis for IHHNV detection. The rest of the shrimp was fixed in Davidson’s alcohol-formalin-acetic acid fixative (Bell and Lightner, 1988) [26] for hematoxylin and eosin (H&E) histology analysis to examine IHHNV-induced pathological changes.

### Histopathology and *in situ* hybridization

Davidson’s alcohol-formalin-acetic acid-fixed samples were processed, embedded in paraffin, and sectioned (5 μm thickness) using standard methods [26]. After staining with H&E, the sections were analyzed by light microscopy. The severity of IHHNV infection/lesion was graded from G0-G4 according to Lightner (Lightner 1996) [2], with G0 being the absence of lesions and G4 being the presence of severe lesions and advanced tissue destruction.

Probes used for *in situ* hybridization (ISH) contained a mixture of digoxigenin (DIG)-labelled probes made using PCR DIG Probe synthesis kit (Roche), and oligonucleotide 3′ end-labeling (Sigma-Aldrich, USA). The IHHNV primers 309F: 5′-TCC AAC ACT TAG TCA AAA CCA A[DIG]-3′ and 309R: 5′-TGT CTG CTA CGATGA TTA TCC A[DIG]-3′ were used for 3′-end labeling. Davidson’s fixative-fixed shrimp were processed, and tissue sections (5 μm thickness) were prepared [20]. After deparaffinization, hydration, proteinase K digestion, and pre-hybridization, the sections were overlaid with 500 μL of hybridization solution containing DIG-labeled primers (100 fmol). The slides were placed on a heated surface at 90°C for 10 min and hybridized overnight at 50°C. Final detection was performed with an anti-digoxigenin antibody conjugated to alkaline phosphatase (Roche), which was visualized using nitro blue tetrazolium and 5-bromo-4-chloro-3-indolyl phosphate [27].

### Statistical analysis

One-way analysis of variance (ANOVA) followed by Tukey’s test for unequal n *post-hoc* mean comparisons was performed to determine the difference in survival between experimental groups. The DNA copy number was expressed in log_10_ and subjected to statistical analysis using Shapiro-Wilk normality test and non-parametric Mann Whitney test with 95% CI comparing IHHNV-infected *P*. *vannamei* vs. *P*. *monodon* vs. *P stylirostris*. The correlation between IHHNV load and prevalence was analyzed through nonparametric correlation analysis using Kendall’s tau-b and Spearman’s rho (SPSS v16).

## Results

### Farm analysis

*Penaeus vannamei* samples collected from grow-out ponds in three different regions of Ecuador, including El Oro, Esmeraldas, and Guayas, were screened for IHHNV using real-time PCR following the OIE-recommended protocol. The details of the samples collected from these regions, IHHNV prevalence, and viral load data are summarized in Table 2.

**Table 2:** Number of *Penaeus vannamei* shrimp per analyzed pond, average weight (g), standard deviation (SD), and coefficient of variation CV (%).

Region	Pond	Number of shrimp	Weight (g) mean ± SD	CV (%)	IHHNV prevalence (%)	Mean IHHNV copy number ngDNA^-1^
Guayas	1	30	10.0±1.7	17.2	13.3	8.02E+00
El Oro	2	30	11.5±3.4	29.3	93.3	4.54E+03
Esmeraldas	3	30	6.2±3.3	52.7	90.0	1.43E+04
Esmeraldas	4	30	7.6±1.9	24.3	6.7	3.95E+01
Guayas	5	30	21.0±3.8	18.1	100.0	1.04E+04
Guayas	6	30	22.5±3.1	13.6	90.0	3.23E+01
Guayas	7	30	25.6±3.7	14.4	50.0	4.12E+03
Guayas	8	30	28.6±3.1	11.0	3.3	5.96E+00

In total, 240 shrimp from eight grow-out ponds were analyzed. Average shrimp size per pond varied between 6.2 and 28.6 g. The coefficient of variation ranged from 13.6% to 52.7%. IHHNV was present in all the grow-out ponds, with a prevalence ranging from as low as 3.3% to 100% (Table 2). There was a significant correlation between IHHNV copy number and IHHNV prevalence (%) (R^2^ = 0.32, p < 0.01).

**Fig 2:**
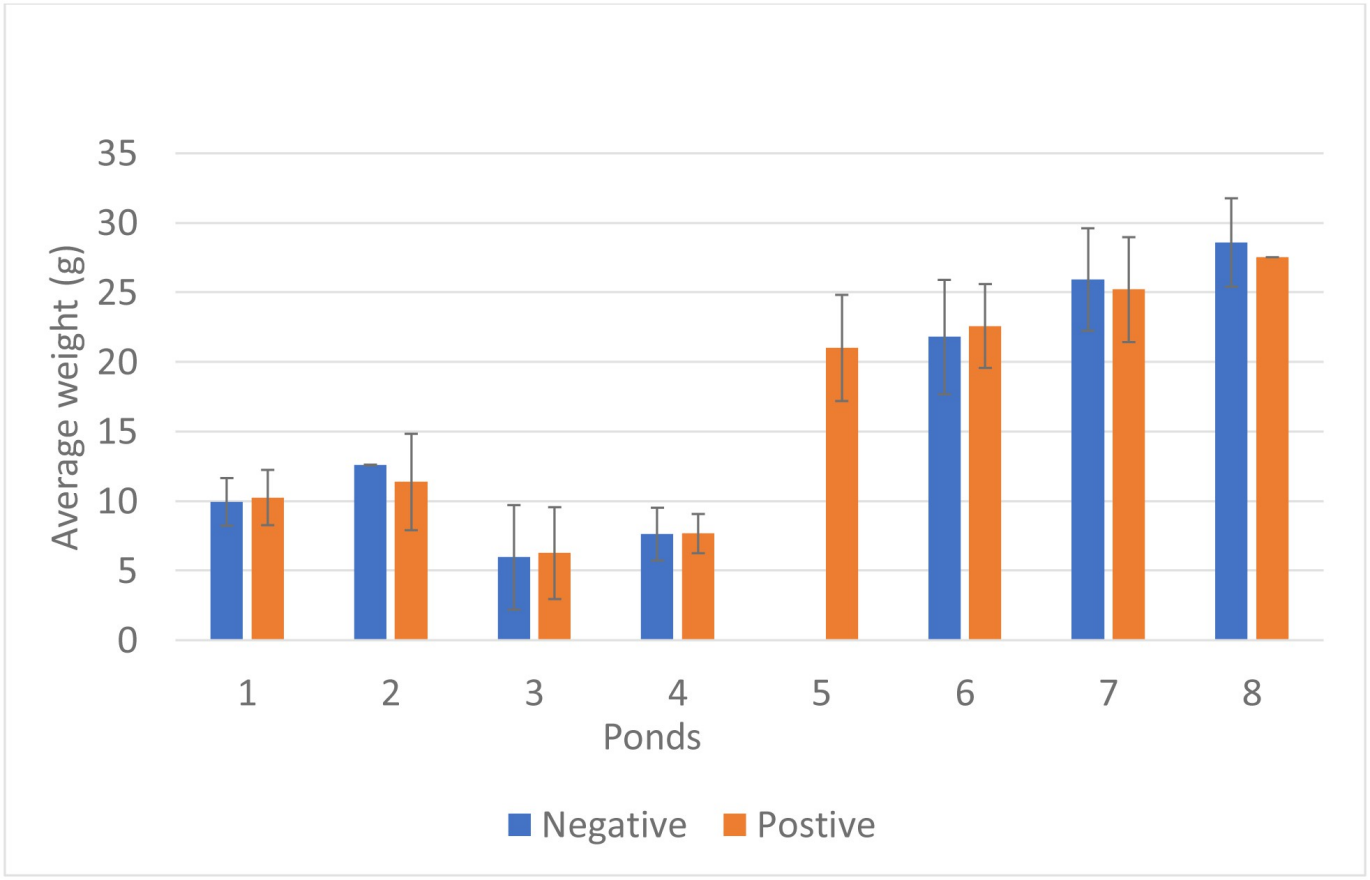
Comparison of average shrimp weight vs. IHHNV status (positive / not detected) in eight grow-out ponds in three regions in Ecuador. Error bars represent the standard deviations.

**Fig 3:**
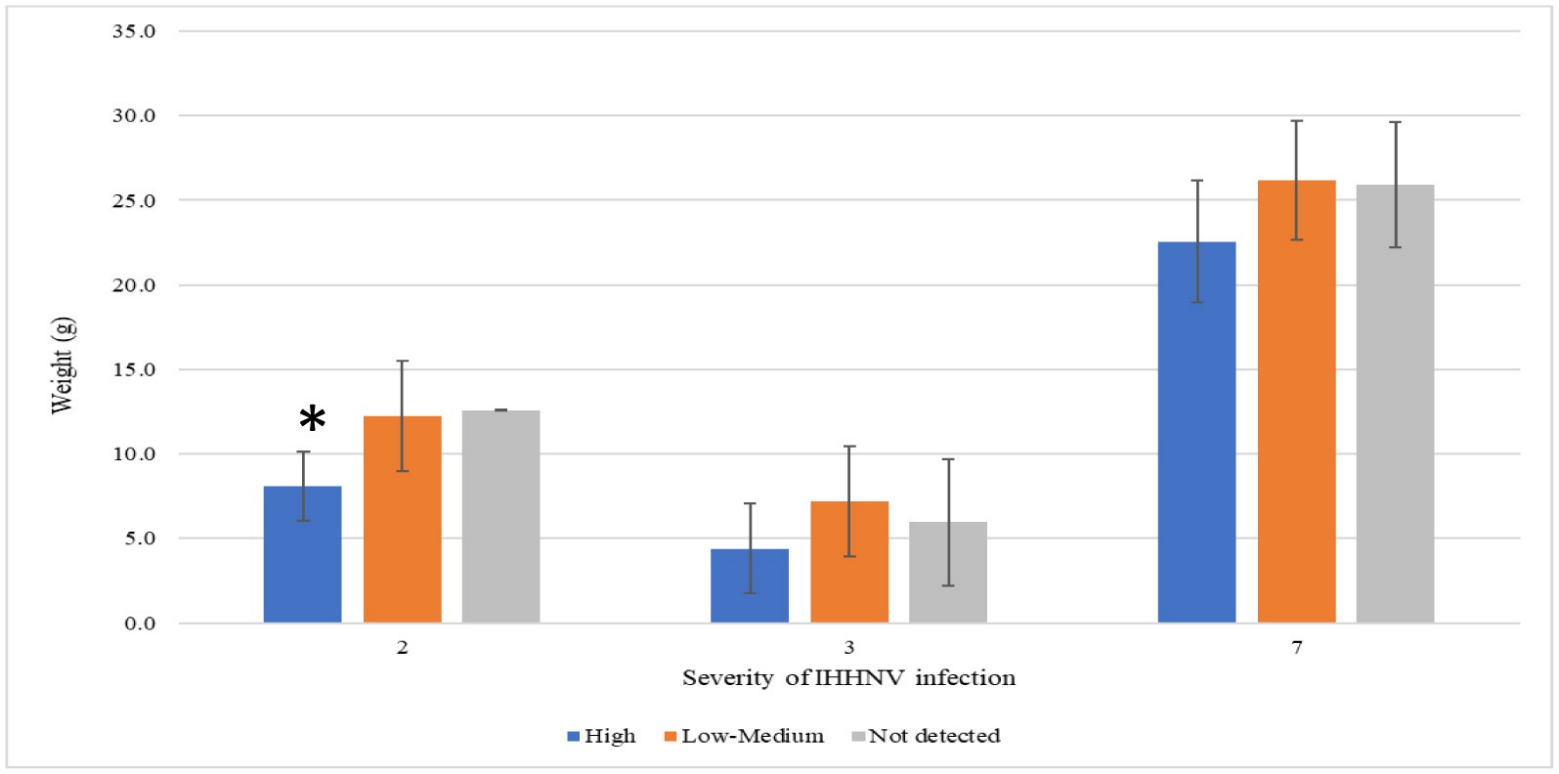
Comparison of IHHNV load (high, low to medium, and not detected) vs. average weight of *Penaeus vannamei* in grow-out ponds 2, 3, and 7 in Ecuador. “*” represents a significant difference by one-way ANOVA (P<0.05).

In the eight grow-out ponds analyzed, there was no significant correlation between the average weight of shrimp in a given pond and the IHHNV prevalence in the corresponding pond (p>0.05) (Fig 2). The IHHNV copy number was calculated for each sample to determine whether there was any correlation between the viral load and animal weight in a pond. To accomplish this goal, IHHNV load was divided into three arbitrary categories: not detected, low-to-medium copies (IHHNV ≤1 × 10^2^ copies/ng DNA), and high copies (>1 × 10^2^ copies/ng DNA). It is important to highlight that not all grow-out ponds had shrimp in all three categories. Therefore, only three ponds (ponds 2, 3, and 7) that contained shrimp representing all three categories were used for this analysis (Fig 3).

The three ponds that had shrimp in all three categories showed a negative trend in shrimp weight vs. IHHNV load. In one of the grow-out ponds (pond 2), there was even a significant difference in average weight (p<0.05) between shrimp with a high IHHNV load (8.1±2.0 g), shrimp with low-to-medium IHHNV load (12.3±3.2 g), and shrimp with no IHHNV detected (12.6±0.0 g) (Fig 3).

### Histopathology and *in situ* hybridization

Of the 72 shrimp from the eight grow-out ponds that were analyzed for H&E histology, only seven presented typical IHHNV lesions. These lesions are characterized by the presence of intranuclear eosinophilic Cowdry type A inclusions (CAI’s) with chromatin marginated in cells from the cuticular epithelium, heart epicardium, and connective tissue. In all cases, the severity of IHHNV infection was low, and the severity scale ranged between G-trace and G1. There were no cases of shrimp with moderate or severe IHHNV lesions. Interestingly, 3 out of 7 shrimps that showed histological lesions were found in a pond that displayed 100% IHHNV prevalence by real-time PCR (Pond 5).

### Next generation sequencing and mapping assembly

Total genomic DNA isolated from 7 IHHNV-infected samples, 6 from Peru, and 1 from Ecuador was subjected to NGS analysis. A summary of the nucleotide sequence data generated using NGS is presented in Table 3. The curated nucleotide data from all seven isolates almost represented the full-length IHHNV genome. The nucleotide sequence length of these seven IHHNV isolates ranged from 3,726 to 4,122 bases and contained all three open reading frames (ORFs), similar to the reference sequence deposited in the GenBank database (Table 3). These include ORF1 representing non-structural protein 1 (NS-1) containing 663 amino acids (aa), ORF2 encoding an NS-2 protein containing 363 aa, and a capsid protein ORF containing 329 aa.

**Table 3:** A summary of next generation sequencing data of the IHHNV genome from *Penaeus vannamei* shrimp originating in Peru and Ecuador.

Sample	Origin	Total paired reads	Mapped reads	Contig size (nt)	IHHNV length (nt)	Mean coverage (x)	GenBank accession number
A	Peru	93,178,502	2,559,650	3,726	3,726	14,855	OM728639
C	Peru	54,461,472	1,468,337	4,001	3,986	12,795	OM728640
D	Peru	30,777,846	1,068,195	4,122	4,122	69,187	OM728641
E	Peru	35,152,118	9,296	4,297	3,958	272.3	OM728643
F	Peru	9,959,988	16,984	4,260	4,048	509.9	OM728644
G	Peru	123,971,146	666	4,122	3,925	20.1	OM728645
E1	Ecuador	20,893,828	1,789	4,009	3,902	62.6	OM728642

### Phylogeny analysis

**Fig 4:**
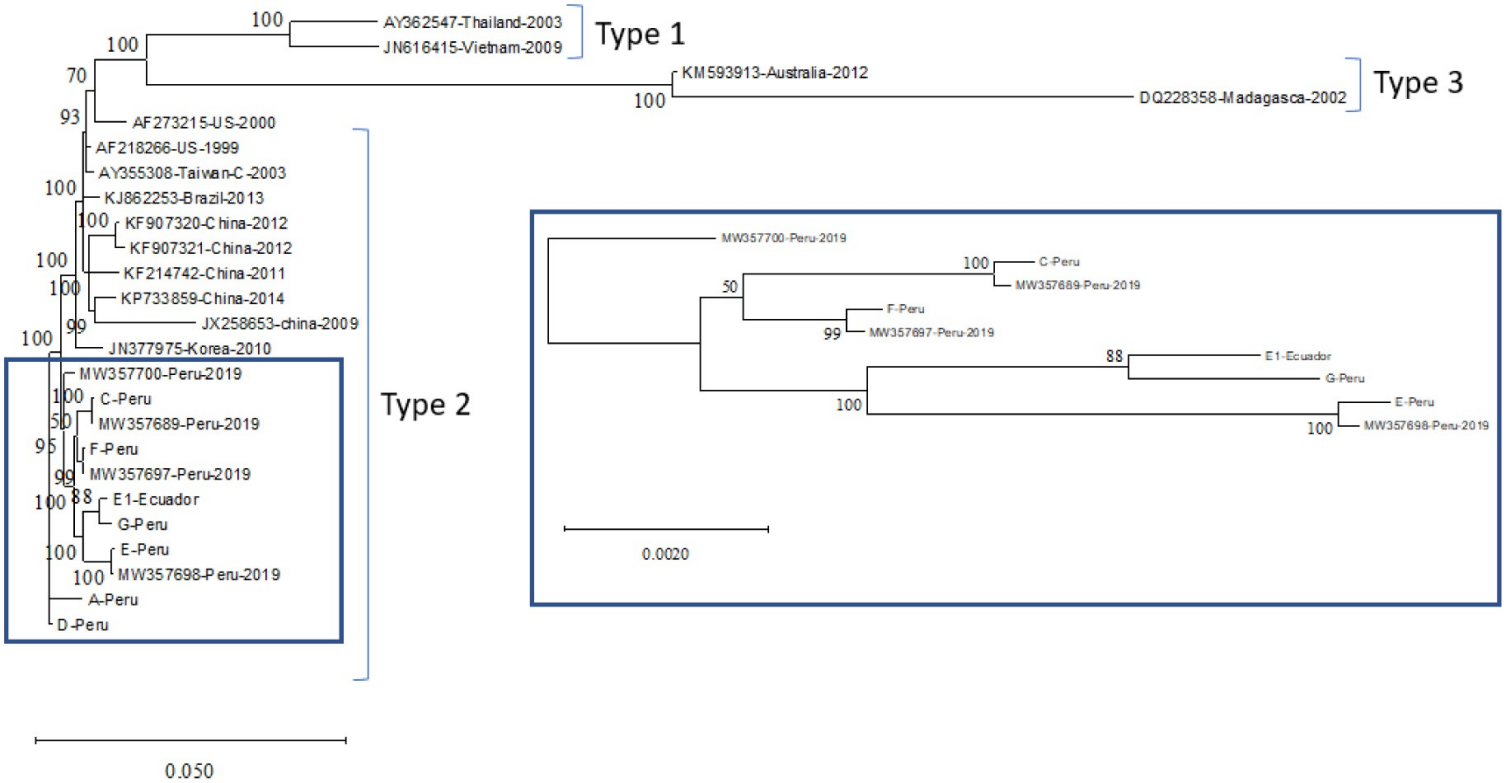
Phylogenetic tree based on nucleotide sequences of IHHNV isolated from Peru (A, C, D, E, F, and G), Ecuador (E1), and other known IHHNV isolates deposited in the GenBank database. Nucleotide sequences were aligned using MAFFT (Auto: according to data size, 200 PAM, k = 2), and the best model for the phylogenetic tree was estimated by jmodel Test 2.1.10 [20]. The phylogenetic tree was built by using MrBayes (http://www.phylogeny.fr/one_task.cgi?task_type=mrbayes) (KY85, 4by4, invariable+Gramma, number of generation = 10,000). The numbers on the tree branches indicate posterior probability. The nucleotide sequences from IHHNV isolated from Peru and Ecuador are shown within a blue box.

**Fig 5:**
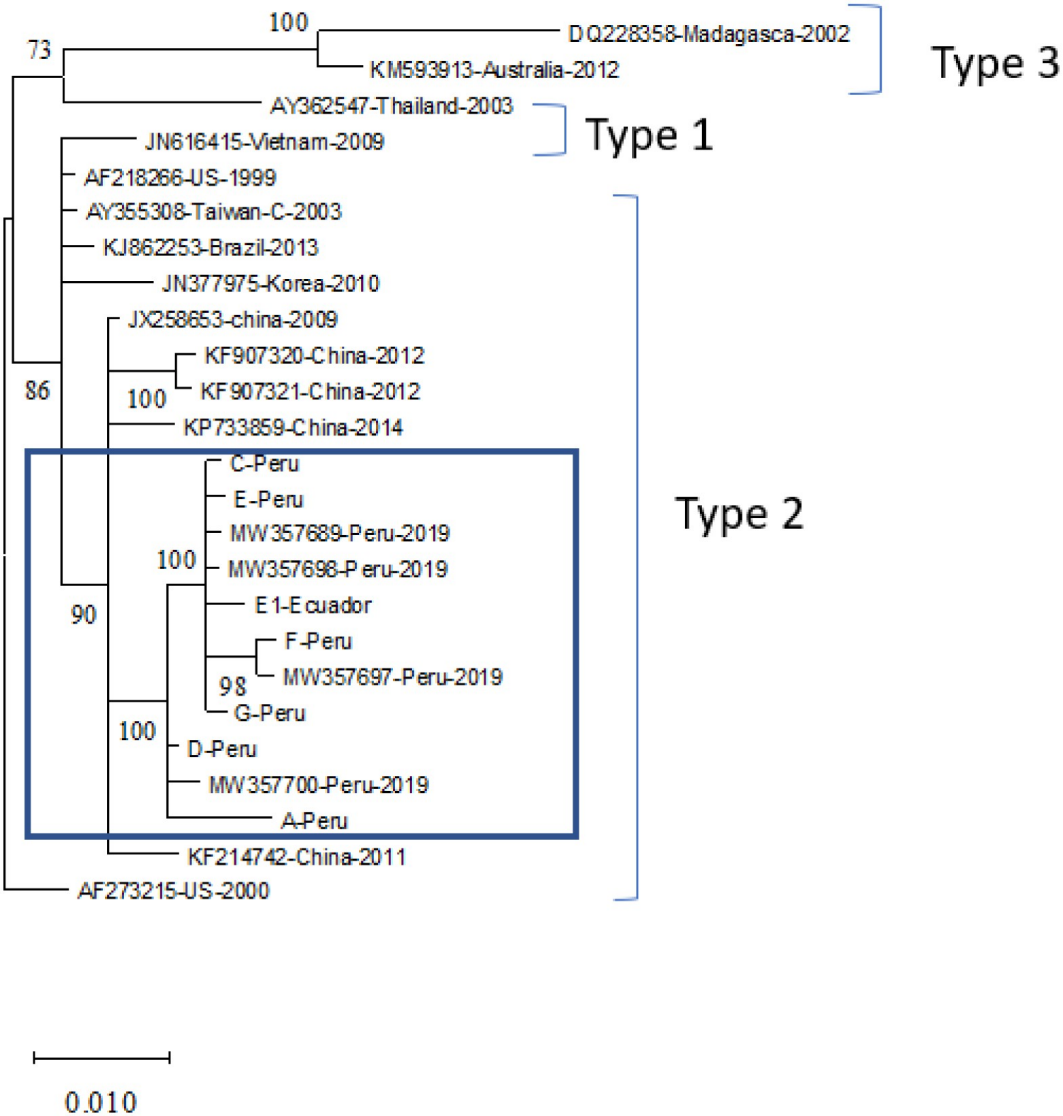
Phylogenetic tree based on the predicted amino acid sequence of capsid protein gene of IHHNV isolates originating in Peru (A, C, D, E, F, and G), Ecuador (E1), and other known homologous IHHNV sequences deposited in the GenBank database. Amino acid sequences were aligned using MAFFT (Auto: according to data size, blossom), and the best model for the phylogenetic tree was estimated by ProtTest 3.4.2 [21]. The phylogenetic tree was built by using MrBayes (http://www.phylogeny.fr/one_task.cgi?task_type=mrbayes) (GTR, VT, no rate variation, number of generations: 10,000). The number on the tree branches indicates posterior probability. The IHHNV isolates from Ecuador and Peru are marked by a blue box.

The whole genome sequence of IHHNV isolates from this study was aligned with 18 known almost full-length IHHNV sequences available in the GenBank database prior to the phylogenetic analysis. The results showed that seven IHHNV sequences clustered with IHHNV isolated from Peru, which was separated from other IHHNV isolates from Latin American countries (Fig 4). Isolates C, E, and F originating in Peru formed a group with the IHHNV isolate previously reported in Peru in 2019, with a posterior probability of 99–100. Isolate G clustered with isolate E1 with a posterior probability of 88. Isolates D and A were obtained from other Peruvian isolates. Likewise, the phylogenetic tree constructed based on the deduced amino acid sequence of the capsid protein also showed that the IHHNV from Peru and Ecuador formed a clade that was separated from the IHHNV previously isolated from the Latin American region (Fig 5). In addition, the Peru-Ecuador clade was supported by posterior probabilities of 98 and 100 (Fig 5).

### Terminal repeats and palindromic sequence analysis

**Fig 6:**
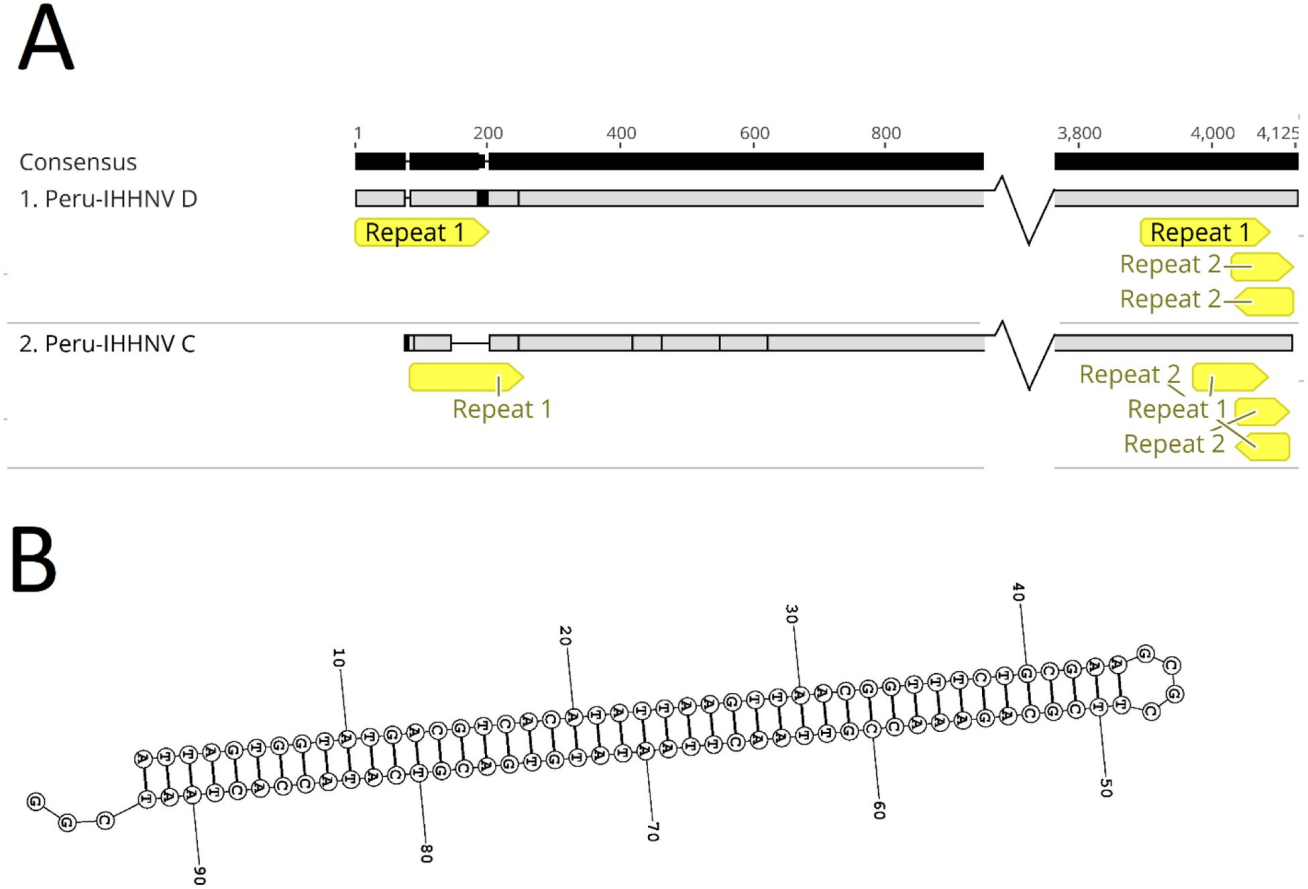
Terminal structures of IHHNV isolates C and D. (A) Direct terminal repeats (repeat 1) located at the 5′ and 3′ ends and the palindromic sequences (repeat 2) located at the 3′ end of the genomes of IHHNV strains D and C. (B) Hairpin model of the palindromic sequences (repeat 2) located at the 3′ end of IHHNV isolate D.

Two out of seven IHNNV genome sequences obtained for this study, isolates C and D originating in Peru, contained DTR at the 5′ and 3′ ends of the viral genome. The DTR1 in isolate D was 197 nt long, with a 99% nucleotide sequence similarity between the 5′- and 3′-end sequences of this repeat (Fig 6A). In IHHNV isolate C, the DTR1 was incomplete and was 114 nt long, with a similarity of 98% between the 5′ and 3′ ends of this sequence. The similarity between the DTR1 of the two IHHNV isolates was 98.4%. Unlike the DTR1, the DTR 2 palindromic sequence in both strains showed a similarity of 100%. The DTR2 in IHHNV isolate D was 12 nt longer (92 nt) than that in IHHNV isolate C (80 nt) (Fig 6A). In addition, repeat 2 formed a well-supported hairpin structure (Fig 6B).

### Pathogenicity of the different IHHNV strains circulating in Peru and Ecuador

Two IHHNV challenges were performed using an isolate from Peru and another isolate from Ecuador. At the end of the IHHNV-challenge, that is, at 39 d.p.i (Peru) and 30 d.p.i (Ecuador), the final survival was high in both experimental challenges. Survival in IHHNV-challenged shrimp was similar to that in the unchallenged group (Table 4).

**Table 4:** Final survival rate in *Penaeus vannamei*, *P*. *monodon*, and *P*. *stylirostris* following IHHNV challenge via injection at 39 (Peru) and 30 (Ecuador) days post-challenge.

Challenge	Origin of the IHHNV isolate	Species	Combined final survival (%)	Prevalence of IHHNV by real-time PCR	Prevalence of IHHNV by H&E and confirmed by ISH
1	Peru	*P*. *vannamei*	100	100% (20/20)	95%
2	Ecuador	*P*. *vannamei*	100	100% (15/15)	86.6%
		*P*. *monodon*	97.3	100% (15/15)	6.2%
		*P*. *stylirostris*	97.6	80% (12/15)	0%

**Fig 7:**
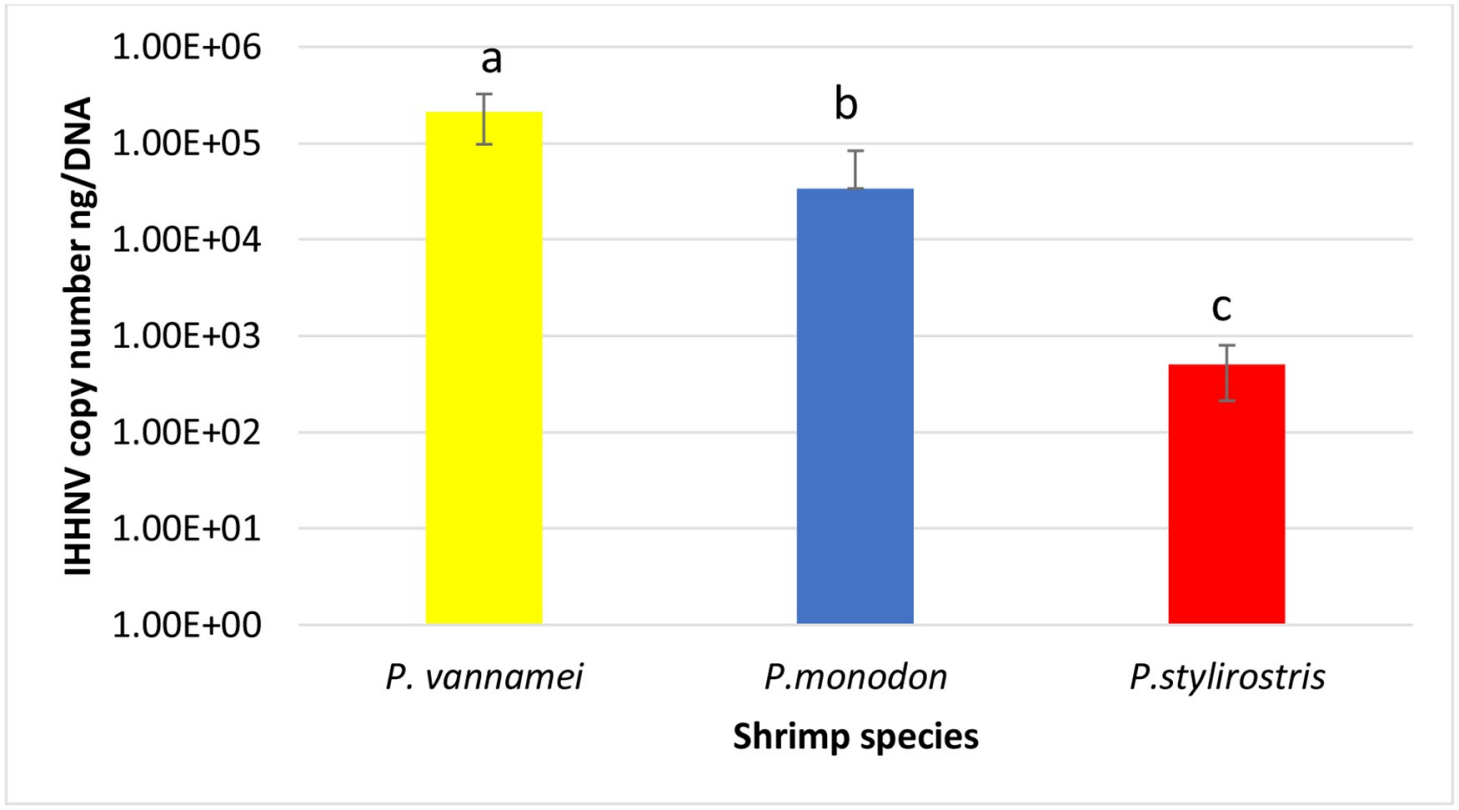
IHHNV copy number in three shrimp populations challenged with IHHNV. Letters a, b, and c denote significant differences by one-way ANOVA (p<0.001).

The final survival rate of *P*. *vannamei* in Challenges 1 and 2 was high (Table 4). In the IHHNV-challenged shrimp, IHHNV infection was analyzed by real-time PCR and histopathology and further confirmed by ISH. Two additional penaeid species, *P*. *monodon* and *P*. *stylirostris* were included in experimental challenge 2. The final survival rates of these two species were also as high as *P*. *vannamei* (Table 4). At the end of the challenge, there was no evidence of RDS or other clinical signs in the IHHNV-challenged shrimp. Real-time PCR confirmed IHHNV in all three species: *P*. *vannamei*, *P*. *monodon* and *P*. *stylirostris*. The IHHNV copy number was highest in *P*. *vannamei* population, followed by *P*. *monodon* and *P*. *stylirostris* (P<0.001) (Fig 7).

**Fig 8:**
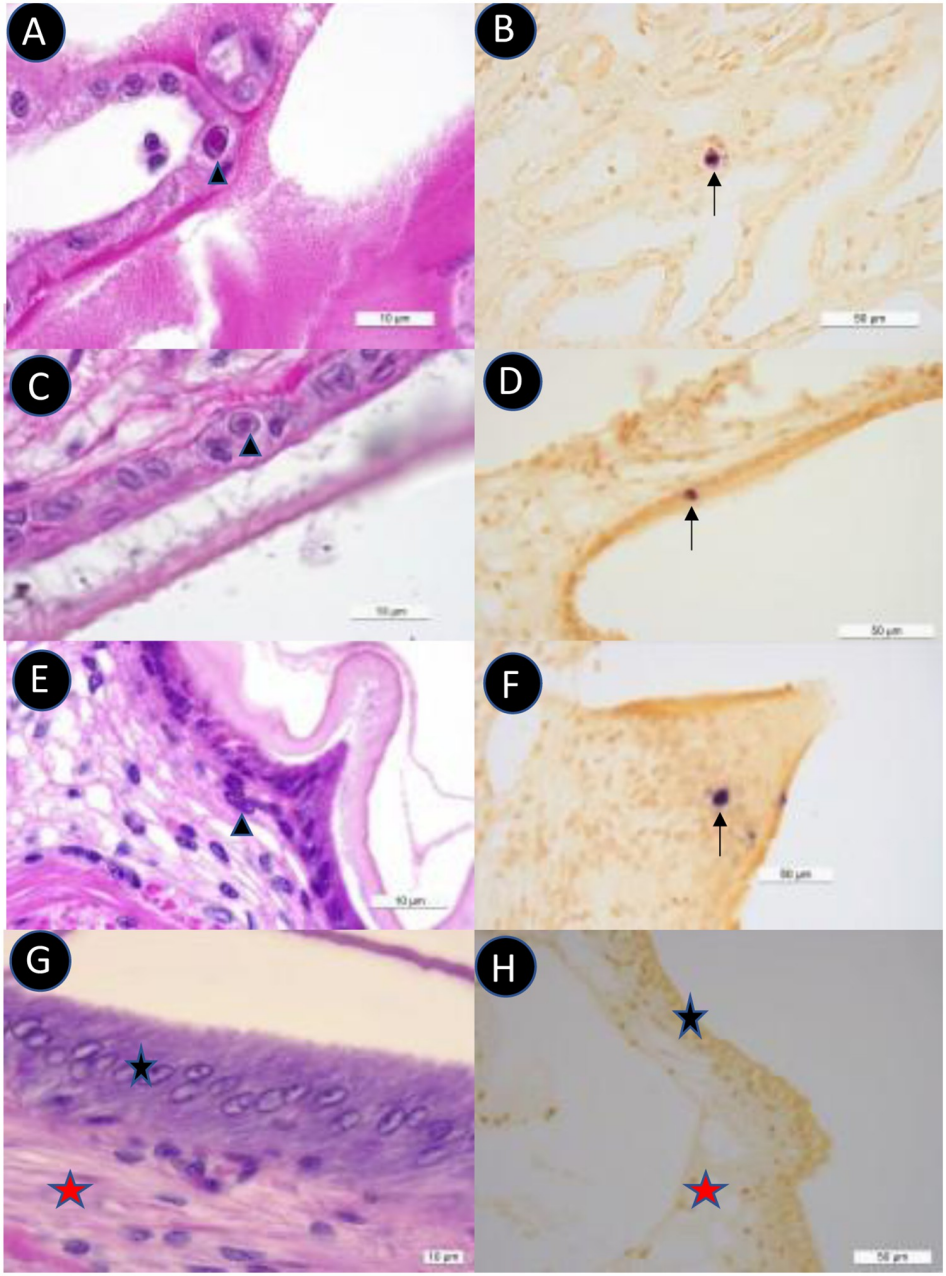
H&E histology and ISH of *P*. *vannamei* and *P*. *monodon* shrimp experimentally challenged with IHHNV. Panels A, C, E, and G represent H&E histology, and Panels B, D, F and H represent ISH results using IHHNV-specific probes. A&B: *P*. *vannamei* infected with IHHNV isolate from Peru, C&D: *P*. *vannamei* infected with IHHNV isolate from Ecuador, E&F: *P*. *monodon* infected with IHHNV isolate from Ecuador, G&H: *P*. *vannamei* Specific Pathogen Free (SPF) control shrimp. A & B shows a section of the antennal gland. C-H show sections of cuticular epithelium. Arrowheads show the eosinophilic intranuclear inclusion body (Cowdry type A). Arrows show a positive reaction by ISH (dark precipitate). The black star represents a section of cuticular epithelium. The blue star shows a section of spongy connective tissue.

To confirm IHHNV infection in experimentally challenged penaeid shrimp, H&E histology and ISH were performed using IHHNV-specific probes. In Table 4, the H&E histology results and ISH data are presented. IHHNV infection was confirmed by H&E histology and ISH in *P*. *vannamei* and *P*. *monodon* in several tissues and organs, including the cuticular epithelium of the gill filaments and antennal gland, nerve cord, lymphoid organ, and epicardium. In contrast, there was no evidence of IHHNV lesions either by H&E histology or ISH in *P*. *stylirostris*. In Fig 8, typical IHHNV intranuclear inclusion bodies are shown by H&E histology and confirmed by ISH in *P*. *vannamei* and *P*. *monodon* in IHHNV-challenged shrimp.

## Discussion

Here, we present data on the prevalence of IHHNV and its impact on the growth of *P*. *vannamei* in grow-out ponds in three major shrimp-producing regions in Ecuador. Furthermore, we determined the genome sequence of the virus in *P*. *vannamei* originating in Ecuador and its neighboring country, Peru, where IHHNV is prevalent. Finally, we performed an experimental bioassay using IHHNV isolates originating from Ecuador and Peru to determine the virulence of the virus on three SPF penaeid shrimp species: *P*. *vannamei*, *P*. *monodon*, and *P*. *stylirostris*.

### Farm analysis

*Penaeus vannamei* samples from grow-out ponds representing three different regions in Ecuador were screened for IHHNV. The virus was present in samples collected from all eight grow-out ponds located in the three different regions (Table 1). The IHHNV prevalence varies widely, from 3.3% up to 100%. In real-time PCR analysis, some shrimp showed negative results, whereas others showed a copy number of up to 2.9 × 10^5^ copies/ng DNA (Table 2). To establish the association between shrimp weight and the presence of IHHNV, the population from each pond was divided into two groups based on real-time PCR results (IHHNV-positive and IHHNV not detected). As shown in Fig 2, there were no significant correlations between the average shrimp weight and presence of IHHNV within the same pond. Sellars et al. [4] showed that in a *P*. *monodon* farm in Australia, growth performance and survival were reduced in grow-out ponds with a high copy number of IHHNV (0.5 × 10^3^ to 2.24 × 10^6^ copies/ng TNA). In our study, the mean copy number of IHHNV varied between 5.9 × 10^0^ and 1.4 × 10^4^ copies/ng DNA, which is lower than the IHHNV load reported by Sellars et al. [4], which may partially explain the little to no influence on growth performance. There was a significant direct correlation between the IHHNV copy number and the prevalence of IHHNV in the grow-out ponds. The prevalence and copy number was high. Similar results in grow-out ponds were reported by Sellars et al. [4]. These findings are consistent with the pattern of infection with infectious agents. Walker et al. [28] suggested that when viral loads increase to acute levels, they can compromise shrimp health. In our study, histological IHHNV lesions were observed only in shrimp from grow-out ponds where the prevalence of IHHNV was 100%. In contrast, shrimp from ponds with a low prevalence of IHHNV did not display any histological lesions.

To better understand the effect of IHHNV load on the growth of *P*. *vannamei* shrimp in a given pond, shrimp populations were divided into three categories based on the IHHNV copy number: not detected, low to medium, and high. Similar criteria were used by Sellars et al. to assess the impact of IHHNV on *P*. *monodon* growth [4]. Only three out of eight grow-out ponds (ponds 2, 3, and 7) had shrimp representative of all three categories. As described in Fig 3, there was an inverse trend between the IHHNV copy number and shrimp weight, with animals having higher viral loads and lower weights. In one case (pond 2), a significant correlation was observed. These grow-out ponds had the highest mean copy numbers of IHHNV. For example, the average IHHNV copy numbers in animals collected from ponds 2, 3, and 7 were 4.54 × 10^3^ copies/ng DNA, 1.43 × 10^.4^ copies/ ng DNA, and 4.12 × 10^3^ copies/ng DNA, respectively (Table 2). One grow-out pond (pond 5) that also showed a high IHHNV mean copy number was not analyzed owing to the fact that 100% of the shrimp tested positive for IHHNV. It is worth noting that RDS was not observed in shrimp collected from any of the three ponds or the remaining five ponds.

Historically, IHHNV was reported to cause RDS in *P*. *vannamei*, resulting in large size disparities and growth deformities [29]. A recent report from India documented deformities including deformed sixth abdominal segment, deformed rostrum, cuticular roughness, and wrinkled antennae, and a wide variation in the size of grow-out ponds in *P*. *vannamei* analyzed from 350 shrimp farms [5]. Unfortunately, in that study, only shrimp with clinical signs were analyzed; therefore, it was not possible to determine IHHNV prevalence in healthy individuals from the same ponds. Another report from India [12] reported the presence of two pathogens, White spot syndrome virus (WSSV) and IHHNV, without any external deformities. From this perspective, our findings suggest a unique first-glimpse of the inherent ability of certain genetic lines of *P*. *vannamei* to carry a high viral load without displaying any clinical signs.

### IHHNV genome sequence and phylogenetic analyses

In this study, the full-length IHHNV genome sequence (AF218266) was used as a reference for mapping. The IHHNV genome varies in length; for example, the IHHNV isolated from Brazil is 3739 nt in length [30], and the IHHNV isolated from India is 3,908 nt in length [31] or even 4,1 kb [9]. In this study, the IHHNV genome had an average size of 3,957 nt, which was in the range of the IHHNV genomic size. The IHHNV genome contains three major ORFs encoding 666 aa-NS1, 363 aa-NS2, and 329 aa-Capsid proteins [31, 32]. The phylogenetic tree constructed from the full genome sequence of IHHNV revealed that all IHHNV isolates from Peru and Ecuador belonged to the same clade type 2 IHHNV group as IHHNV isolates from East Asia and the Americas, as described elsewhere (Dhar et al., 2019). However, it seems that they form a new sub-clade within this type 2 strain. Indeed, the phylogenetic tree built from CP protein sequences also revealed a unique sub-clade containing all IHHNV from Peru and Ecuador. Therefore, we propose a new type of IHHNV isolated from these two geographical regions. One possible explanation is the frequent movement of post-larvae from Ecuador to Peru. During active surveillance conducted by SANIPES, samples of imported post-larvae from Ecuadorian lots were analyzed by PCR. The frequency of IHHNV in these samples was 55% (15 out of 27 samples) in 2019, 52% (75 out of 144 samples) in 2020, and 76.3% (55 out of 72 samples) in 2021 [33].

### Identification of terminal repeats and palindromic sequences in the IHHNV genome

We identified DTR at the 5′ and 3′ ends of the IHHNV (strains C and D) genome, and the DTR at the 3′ end contained palindromic sequences that formed putative hairpin-like structures that are hallmarks of the *Parvoviridae* family [34]. As observed in HPV, only the 3′ end sequences were able to form putative hairpin-like structures [35] and unlike in other shrimp parvoviruses, in *P*. *monodon* metallodensovirus, we did not observe a T-shaped structure in the inverted terminal repeats [7]. This demonstrates the uniqueness of the novel IHHNV sequences found in Latin America.

### Virulence of IHHNV isolates from Ecuador and Peru determined by experimental challenge

For the experimental challenge, the IHHNV-Ecuador isolate, an IHHNV isolate from Peru, was used to experimentally infect SPF shrimp. The virulence of the IHHNV isolates was determined based on the final survival rate, RDS, IHHNV copy number, histological lesions, and ISH. Although SPF *P*. *vannamei* was used for the IHHNV bioassays for both Ecuador and Peru isolates, two additional species, *P*. *monodon* and *P*. *stylirostris*, were tested for the IHHNV-Ecuador bioassay. Overall, the survival was very high in all three species (97–100% survival), even when the inoculum was administered via injection with a high viral copy number (9.0 × 10^6^ copies). These results clearly support the notion that these SPF lines are tolerant to the IHHNV isolates used in the bioassay. Despite the lack of mortality in the bioassay, IHHNV-injected shrimp had a high IHHNV load at the end of the experiment. The IHHNV strains from Peru and Ecuador did not cause mortality in two endemic shrimp species (*P*. *vannamei* and *P*. *stylirostris*) and one exotic shrimp species (*P*. *monodon*). These findings can be explained in part by considering the historical background of the *P*. *stylirostris* stock. The genetic line of *P*. *stylirostris* used in this study originated from Mexico, and is likely a progenitor of an IHHNV-resistant stock that was selected from survivors of an IHHNV outbreak. Around 1999, there was a line of *P*. *stylirostris* called “Super Shrimp^®^” that was confirmed to be resistant to IHHNV after the IHHNV challenge test for 32 d.p.i without any evidence of harboring IHHNV, as assessed by TaqMan real-time PCR [21]. In 2010, this genetic line was obtained from Mexico to Hawaii, where it was maintained for approximately 10 years in facilities with high biosecurity measures. This genetic stock appeared to retain the tolerance/resistance to IHHNV.

**Fig 9:**
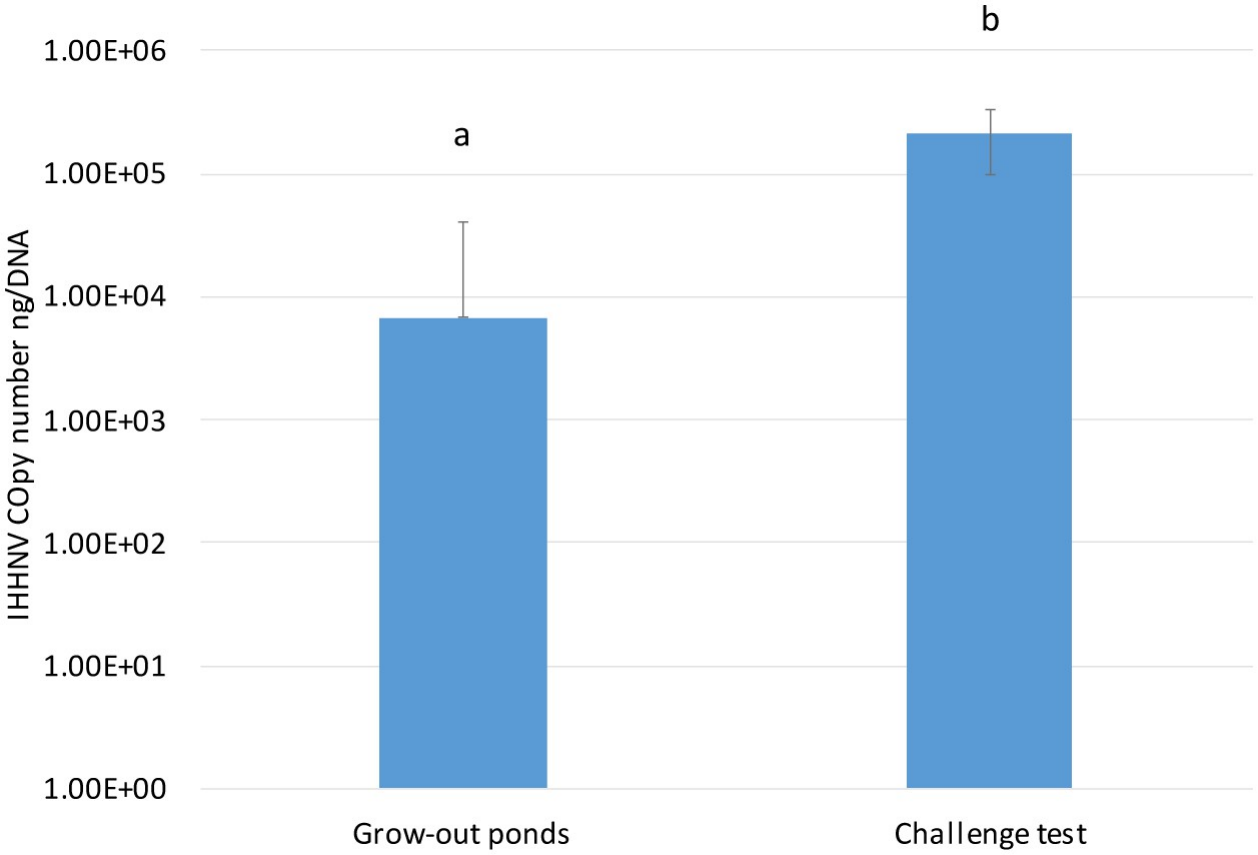
IHHNV copy number in a SPF *P*. *vannamei* line experimentally challenged with IHHNV vs. copy number in 240 *P*. *vannamei* shrimp from eight grow-out ponds in Ecuador. Letters a and b denote significant differences by one-way ANOVA (p<0.001).

When the IHHNV load in *P*. *vannamei* raised in grow-out ponds in Ecuador was compared to the IHHNV load found in the SPF *P*. *vannamei* used in the experimental challenge, there was a significant difference in the viral loads (P<0.001), with a higher IHHNV copy number in the SPF population used for the challenge test than in the farm-raised *P*. *vannamei* (Fig 9). Although the genetic backgrounds of the SPF line used in the bioassay and the farm-raised *P*. *vannamei* are unknown, the data suggest a high tolerance of endemic shrimp populations that are being raised in the Ecuadorian shrimp industry. Currently, in Latin America, most shrimp have been raised under conditions where IHHNV is endemic, and the use of pond-rear broodstock has apparently acquired unintentional selection for resistance to diseases, including IHHNV [36]. This tolerance/resistance may explain the absence of RDS associated with IHHNV infection in commercial grow-out ponds rearing *P*. *vannamei*.

In the shrimp industry in Latin America, IHHNV has been one of the most prevalent pathogens reported in the last 20 years by the World Animal Health Information System (WAHIS) [11]. Viral accommodation is a hypothesis proposed earlier in 2009 and then updated in 2019 [37, 38]. It is related to tolerated persistent infection where viruses or fragments of their genome are inserted into the shrimp genome by an autonomous host mechanism that might have a protective effect, which could explain the absence of clinical signs. Furthermore, Taengchaiyaphum [19] recently confirmed a significant reduction in the IHHNV copy number in *P*. *vannamei* where IHHNV circular viral copy (cvcDNA) was present. This phenomenon could explain the absence of RDS and severe histological lesions in shrimp infected with IHHNV in Latin America.

## Conclusions

IHHNV is a pathogen with high prevalence in shrimp farms in Latin America. We surveyed the prevalence of IHHNV in commercial grow-out ponds in three major shrimp-producing regions of Ecuador and assessed its impact on shrimp growth. Upon confirming the presence of the virus in commercial ponds in Ecuador, we determined the genome sequence of the virus in its endemic range from Ecuador and Peru. Finally, using an experimental bioassay, we determined the virulence of the IHHNV-Ecuador and -Peru isolates using three different penaeid shrimp species: *P*. *vannamei*, *P*. *monodon*, and *P*. *stylirostris*. Our data revealed that IHHNV did not cause mortality in the challenged species, and did not have a noticeable impact on shrimp production.

A wide variation in IHHNV prevalence is likely due to many factors, such as the days of culture when the samples were collected, health status of the post-larvae used to stock the ponds, genetic background of the post-larvae, culture conditions, farm management, and other unknown factors. Leaving aside the percent prevalence data, in ponds with animals carrying no IHHNV to high levels of IHHV, we found a negative trend between the IHHNV load and shrimp weight in the corresponding pond. NGS data revealed that circulating IHHNV genotypes in Ecuador and Peru represent the infectious form of IHHNV. Finally, in laboratory bioassays that lasted 30 days using IHHNV isolates from Ecuador and Peru, we could not reproduce any clinical manifestation of IHHNV infection in *P*. *vannamei* or any significant mortality in any of the three penaeid shrimp species. In addition, although IHHNV was detected in all three species using real-time PCR, the viral load was significantly higher in *P*. *vannamei* than in *P*. *monodon* and *P*. *stylirostris*. Moreover, using H&E histology analysis, Cowdry type A inclusion bodies, which are considered pathognomonic of IHHNV infection, were detected in *P*. *vannamei* and *P*. *monodon*, but not in *P*. *stylirostris*. ISH was performed to delineate the IHHNV-specific lesions in *P*. *vannamei* and *P*. *monodon*.

Although our data on IHHNV quantitative load and its effect on growth are limited, they suggest a negative correlation between these two parameters. Therefore, a more robust data set with a larger number of samples coupled with the health status of the post-larvae used to stack a pond will be immensely beneficial for a more accurate investigation of a quantitative relationship between shrimp growth and pathogen load of circulating IHHNV genotypes in Latin America.
